# Ionic liquid‐based dispersive liquid–liquid microextraction of anthelmintic drug residues in small‐stock meat followed by LC‐ESI‐MS/MS detection

**DOI:** 10.1002/fsn3.3568

**Published:** 2023-07-22

**Authors:** Rebagamang Tshepho, Simiso Dube, Mathew M. Nindi

**Affiliations:** ^1^ Department of Chemistry, College of Science, Engineering and Technology, The Science Campus University of South Africa Roodepoort, Johannesburg South Africa; ^2^ Residue Section Botswana National Veterinary Laboratory Gaborone Botswana; ^3^ Institute for Nanotechnology and Water Sustainability (iNanoWS), The Science Campus, College of Science, Engineering and Technology (CSET) University of South Africa Roodepoort, Johannesburg South Africa

**Keywords:** anthelmintic drugs, caprine, green sample preparation, ionic liquid‐dispersive liquid–liquid microextraction, ovine

## Abstract

An ionic liquid‐based dispersive liquid–liquid microextraction (IL‐DLLME) of 20 anthelmintic drugs followed and detected by liquid chromatography–tandem mass spectrometry (LC–MS/MS) has been developed, optimized, and validated. The parameters affecting the anthelmintic extraction efficiencies such as selection of extraction solvent (ionic liquids), selection of disperser solvent, volume of extraction solvent, volume of disperser solvent, pH of the aqueous phase, extraction time, salt addition, and centrifugation time were optimized. Validation was conducted according to ISO/IEC 17025:2017 and Commission Implementing Regulation (EU) 2021/808 of 22 March 2021. Validation parameters such as calibration function, matrix effect, limit of detection (LOD), limit of quantification (LOQ), decision limit (CCα), accuracy, and precision were established. Coefficient of determination (*R* ^2^) values ranging from .99938 to .99995 were obtained using the matrix calibration curve spiked at 0, 0.25, 1.0, 1.5, and 2.0 times MRL. The LODs and LOQs were calculated using the standard deviation of the response and the slopes of the calibration curves ranged from 0.35 to 26.1 μg/kg and from 1.2 to 87.0 μg/kg, respectively, and were dependent on calibration range. The CCα values ranged from 23 to 1022.0 μg/kg and are also dependent on the MRL concentration levels. The coefficient of variation (CV) values calculated are within the reproducibility range of 16%–30% adapted from the Horwitz Equation CV = 2^(1–0.5 log C)^ and ranged from 1.7% to 16.9%. The developed and validated and the standard QuEChERS method were compared. The IL‐DLLME LC–MS/MS method was applied to 32 small stock (18 caprine [goat] and 14 ovine [sheep]) liver samples received from municipal abattoirs at Botswana National Veterinary Laboratory for the analysis of anthelmintic drug residues. The results obtained indicated that the anthelmintic drug residues were all below the detection capability, and therefore, the samples were passed as fit for human consumption.

## Graphical abstract

The work is on development and validation of a green sample preparation method for the extraction of anthelmintic drugs from caprine and ovine.

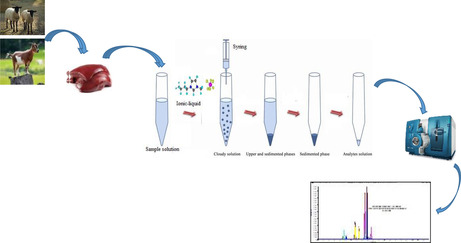

## 1 INTRODUCTION

Anthelmintic drugs are used to treat livestock and humans infected with parasitic worms or helminths (helminthiasis). These drugs are classified based on similar chemical structures or mode of action. They are classified as benzimidazoles, probenzimidazoles, salicylanilides, macrocyclic lactones, imidazothiazoles, tetrahydropyrimidines, spiroindoles, and amino‐acetonitrile derivatives. Benzimidazoles are the largest chemical family of anthelmintics used in domestic animals. They are grouped into benzimidazole thiazoles which include thiabendazole, cambendazole, etc., methyl benzimidazole carbamates (parbendazole, mebendazole, flubendazole, oxibendazole, luxabendazole, albendazole, albendazole sulfoxide, fenbendazole, oxfendazole, etc.), halogenated benzimidazole thiol derivatives (triclabendazole, triclabendazole sulfone, triclabendazole sulfoxide, etc.), and pro‐benzimidazoles (thiophanate, febantel, and netobimin). Some anthelmintic drugs act rapidly and selectively on neuromuscular transmission of nematodes. Levamisole, pyrantel, and morantel are agonists at nicotinic acetylcholine receptors of nematode muscle and cause spastic paralysis. Salicylanilides, which include rafoxanide, oxyclozanide, brotianide, and closantel, and the substituted phenol, nitroxinil, are proton ionophores (compounds that facilitate transmembrane transport of protons). Clorsulon is a selective antagonist of fluke phosphoglycerate kinase and mutase (Argüello‐García et al., 2020; De Graef et al., 2013; Dyary, 2015; Enejoh & Suleiman, 2017; Holden‐Dye & Walker, 2007, 2014; Martin, 1997; Singh et al., 2012). The intensive use or abuse of anthelmintic drugs can lead to drug resistance. It is very difficult to control helminths carrying drug‐resistant genes since they produce drug‐resistant larvae and increase the population of resistant parasites in the environment (De Graef et al., 2013; Dyary, 2015; Gioti et al., 2005). In addition to challenges of drug residue in meat, humans are also exposed to drug‐resistant parasites.

The EU established protocols for testing and monitoring veterinary drugs which involved setting maximum residue levels (MRLs) to ensure that meat destined for local and export markets is safe for human consumption and public health. Anthelmintic drugs (allowed substances) and their maximum residue limits in foodstuff of animal origin are listed in annex of Commission Regulation (EU) No 37/2010 of 22 December 2009 (Commission Regulation, 2010). The MRLs of the marker residues have been set in all ruminant livers for anthelmintic drug as listed in Table 1.

**TABLE 1:** Anthelmintics and their MRLs as in Commission Regulation (EU) No 37/2010.

Anthelmintics	Marker residue	Animal species	Matrix	MRL (μg/kg)
Albendazole	Sum of albendazole sulfoxide, albendazole sulfone, and albendazole 2‐amino sulfone, expressed as albendazole	All ruminants	Liver	1000
Levamisole	Levamisole	Bovine, ovine, porcine, poultry	Liver	100
Oxyclozanide	Oxyclozanide	All ruminants	Liver	500
Rafoxanide	Rafoxanide	Ovine	Liver	150
Fenbendazole	Sum of extractable residues which may be oxidized to oxfendazole sulfone	All ruminants, porcine animals, Equidae	Liver	500
Flubendazole	Sum of flubendazole and (2‐amino 1H‐benzimidazol‐5‐yl) (4‐fluorophenyl) methanone	Porcine, chicken, turkey, game birds	Liver	400
Mebendazole	Sum of mebendazole methyl (5‐(1‐hydroxy,1‐phenyl) methyl‐1Hbenzimidazol‐2‐yl) carbamate and (2‐amino‐1H‐benzimidazol‐5‐yl) phenylmethanone, expressed as mebendazole equivalents	Ovine, caprine, equine	Liver	400
Oxibendazole	Oxibendazole	Porcine	Liver	200
Thiabendazole	Sum of thiabendazole and 5‐hydroxy thiabendazole	Bovine, caprine	Liver	100
Triclabendazole	Sum of extractable residues that may be oxidized to keto‐triclabendazole	Bovine, ovine	Liver	100
Nitroxinil	Nitroxinil	Bovine, ovine	Liver	20

Several techniques, including gas chromatography (GC), high‐performance liquid chromatography (HPLC), and liquid chromatography–mass spectrometry (LC–MS/MS; Bustamante‐Rangel et al., 2022; Guo et al., 2010; Kiltz, 2022a, 2022b; Kinsella et al., 2009; Pavlović et al., 2019; Rawa‐Adkonis et al., 2006), have been used to analyze anthelmintics in a variety of matrices. The GC–MS technique is most appropriate for volatile and nonpolar analytes which makes it limited since most of the anthelmintic drugs are polar and might require laborious derivatization. For this reason, LC–MS/MS has become the preferred method due to its improved sensitivity and selectivity, accuracy, and precision.

The determination of anthelmintic drug residues in biological matrices requires an extraction step that releases analytes from the sample matrix. Complex biological samples contain the analyte alongside a diverse range of chemicals that can have an adverse impact on the accurate and precise quantification of the analyte. Caprine and ovine liver contain protein which may impact the accuracy and precision of quantification. Caprine liver, for example, contains 20.4% of protein and 2.1% of saturated fat, while ovine liver contains about 30.6% of protein and 1.7% of saturated fat (Kiltz, 2022a, 2022b). The most commonly used extraction techniques for the determination of anthelmintic veterinary drug residues are the quick, easy, cheap, effective, rugged, and safe (QuEChERS) method and liquid–liquid extraction (LLE) which are usually followed by cleanup via dispersive solid‐phase extraction (dSPE) and/or solid‐phase extraction (SPE; Gioti et al., 2005; Kinsella et al., 2009; Msagati, 2010; Rawa‐Adkonis et al., 2006). The drawback of the classical LLE method is that it is labor intensive, utilizes large quantities of nongreen organic solvents (creating an environmental challenge), often has low enrichment factors, low selectivity, possible formation of emulsion, and has problems with handling large sample volumes (Rawa‐Adkonis et al., 2006). SPE was developed to overcome a number of LLE drawbacks; however, it also has its own limitations which include the use of large organic solvents, possibility of low recoveries due to interaction between sorbent and sample matrix, low reproducibility due to differences between sorbent batches and sorbent bed clogging (cartridges as well as disks) by particles of sample suspended matter (Rawa‐Adkonis et al., 2006). In the past two decades, a greener sample preparation approach, liquid‐phase microextraction (LPME), emerged as an attractive alternative to the traditional extraction and cleanup methods because of its simplicity, effectiveness, low cost, and small volume of organic solvent requirements. Examples of microextraction techniques included supported liquid membrane (SLM; Msagati & Nindi, 2006), single‐drop liquid‐phase microextraction (SD‐LPME; Gioti et al., 2005), hollow fiber‐based liquid‐phase microextraction (HF‐LPME), and ultrasound‐assisted surfactant‐enhanced emulsification microextraction (UASEME) which has been used in milk formulae and milk samples (Boontongto et al., 2014; Hwang et al., 2018; Mmereki et al., 2014; Payán et al., 2009; Santaladchaiyakit & Srijaranai, 2014; Wang et al., 2015). Amongst these LPME methods is dispersive liquid–liquid microextraction (DLMME) which has not been used previously for anthelmintic drugs but has demonstrated great success for the determination of other veterinary drugs in biological matrices (Mookantsa et al., 2016; Santaladchaiyakit & Srijaranai, 2014). Conventional DLLME uses minute chlorinated organic solvents for extraction. As much as the volumes are in microliters, there is still needed to replace these toxic organic solvents with greener solvents such as ionic liquids (IL). An ionic liquid (IL) is a compound composed of a cation, an anion, and an alkyl, which has low volatility, good extraction properties, and is regarded as a green solvent because of its properties. Due to these properties, the incorporation of ionic liquids as DLLME extraction solvents is more environmentally friendly when compared to other extraction solvents such as chloroform, hexane, and dichloromethane. The IL‐DLLME method was first reported to extract polycyclic aromatic hydrocarbons in water and the sample preparation was successful with good results (Mookantsa et al., 2016; Scheme of the IL‐DLLME; Zgoła‐Grześkowiak & Grześkowiak, 2011). In this study, the focus was on developing an IL‐DLLME extraction method for 20 anthelmintic drugs followed by LC–MS/MS determination. In addition to optimization of all the factors that may affect the efficiency of IL‐DLLME, the developed extraction method was validated in accordance with ISO/IEC 17025:2017 and Commission Implementing Regulation (EU) 2021/808 of 22 March 2021. The IL‐DLLME method was compared with the standard QuEChERS method which is validated and accredited by Southern African Development Community Accreditation Service for ISO/IEC 17025:2017 compliance.

## 2 EXPERIMENTAL PROCEDURE

### 2.1 Reference standards

Reference standards of albendazole (ABZ), albendazole‐2‐amino sulfone (_−_ABZ‐SO_2_ NH_2_), albendazole sulfone (ABZ‐SO_2_), albendazole sulfoxide (ABZ‐SO), fenbendazole (FBZ), 2‐amino flubendazole (FLU‐NH_2_), mebendazole (MBZ), amino mebendazole (MBZ‐NH_2_), hydroxy‐mebendazole (MBZ‐OH), 5‐hydroxy thiabendazole (TBZ‐OH), triclabendazole (TCZ), triclabendazole sulfone (TCZ‐SO_2_), triclabendazole sulfoxide (TCZ‐SO), levamisole (LEVA), clorsulon (CLOR), nitroxinil (NITRO), oxyclozanide (OXY), rafoxanide (RAF), and oxibendazole (OBZ) were obtained from Dr. Ehrenstorfer GmbH (Augsburg, Germany); thiabendazole (TBZ) from Sigma‐Aldrich (Steinheim, Germany); labeled standards flubendazole‐d3 (FLU‐d_3_), mebendazole‐d3 (MBZ‐d_3_), and albendazole methyl‐d3 (ABZ‐d_3_) were obtained from Toronto Research Chemicals; and fenbendazole‐d3 (FBZ‐D_3_) and oxibendazole‐d7 (OBZ‐d_7_) sourced from the EU Reference Laboratory, Berlin were all supplied by LGC Standards GmbH (Wesel, Germany).

### 2.2 Reagents and chemicals

Water for chromatography (LC–MS grade) LiChrosolv®, acetonitrile (ACN) hypergrade for LC–MS LiChrosolv®, methanol (MeOH) hypergrade for LC–MS LiChrosolv®, acetone ACS reagent, formic acid ACS reagent, 1‐butyl‐3‐methylimidazolium hexafluorophosphate ([C4MIM][PF6]) Sigma‐Aldrich HPLC, 1‐methyl‐3‐octylimidazolium hexafluorophosphate ([C8MIM][PF6]) Sigma‐Aldrich HPLC, 1‐hexyl‐3‐methylimidazolium hexafluorophosphate ([C6MIM][PF6]) ROTH HPLC, sodium chloride (NaCl) Rochelle Chemicals, and dimethyl sulfoxide (DMSO) were purchased from Chemoquip (Pty) Ltd and some reagents were donations from IAEA.

### 2.3 Apparatus

A Heraeus (Biofuge Primo) centrifuge, Thermo Scientific (Orbital shaker PSU‐20i), Ultra‐Turrax® (T25 probe homogeniser), Concentration Workstation (TurboVap LV) supplied by Caliper Life Sciences, Analytical Balance ((AS 220/C/2) RADWAG), top pan balances (PGW 253e) Adam, vortex mixer whirl mixer, micropipettes MACROMAN® (0.1, 0.2, 1.0 and 5.0 mL), pH meter inoLab®, ultrasonic bath, and 15‐ and 50‐mL polypropylene centrifuge tubes.

### 2.4 Preparation of stock and working standard solutions

Stock standard solutions (1 mg/mL) for individual compounds were prepared by dissolving each standard in DMSO/MeOH, (50/50) v/v (anthelmintic drug standards dissolve best DMSO and do not precipitate during storage. DMSO freezes fast and takes long time to thaw before use, methanol was added to reduce the thawing time). The prepared stock standard solutions are stored at −20°C. Working standard solution mixture volumes of the stock standard solution (1 mg/mL) for each compound were prepared as follows: albendazole, albendazole sulfoxide, albendazole sulfone, and albendazole‐2‐aminosulfone: 1000 μL to make 10.0 μg/mL; fenbendazole, flubendazole, and oxyclozanide: 500 μL to make 5.0 μg/mL; amine mebendazole, hydroxy‐mebendazole, 2‐amino flubendazole, and mebendazole: 400 μL to make 4.0 μg/mL; triclabendazole, triclabendazole sulfone, and triclabendazole sulfoxide: 250 μL to make 2.5 μg/mL; oxibendazole: 200 μL to make 2.0 μg/mL; rafoxanide: 150 μL to make 1.5 μg/mL; thiabendazole, 5‐hydroxy thiabendazole, levamisole, and clopidol: 100 μL to make 1.0 μg/mL; and nitroxinil: 20 μL to make 0.2 μg/mL. These solutions were transferred to a 100‐mL volumetric flask and filled up to the mark with methanol and stored at −20°C.

### 2.5 Procedure

#### 2.5.1 Isolation of anthelmintic drug residue from liver samples

Isolation of anthelmintic drug residues from liver samples was done by chopping up about 10 g of liver sample and homogenizing the chopped liver in a food processor. Thereafter, 2.0 ± 0.02 g of the previously homogenized liver sample was weighed into a 50‐mL polypropylene centrifuge tube. Three blank samples were fortified with working standard solution at 1.0 MRL (equivalent to 200 μL of working standard) for quality controls. Five‐point pre‐extraction spiked matrix standards (PrEMS), fortified with working standard solution at spiking levels of 0.0, 0.5, 1.0, 1.5, and 2.0 MRL (equivalent to 0, 100, 200, 300, and 400 μL of working standard, respectively), were used for calibration. To all the samples, control samples and calibration samples, 100 μL of stable isotope labeled/internal standard was added. After fortification, the samples were allowed to stand for a minimum time of 30 min before extraction. Using a bottle‐top dispenser, 8 mL of acetonitrile was then added to all the samples. Thereafter, the samples were homogenized for about 20 s using a probe homogenizer and then mixed vigorously on a vortex mixer for 10 min. The tube was then centrifuged at 2795 *g* for 10 min, and the supernatant was collected and concentrated to about 100 μL in a stream of nitrogen in the water bath set at 50°C. The concentrated solution was diluted with 5 mL of water and filtered through 0.45‐μm PVDF syringe filters. The sample was then ready for the IL‐DLLME extraction procedure.

#### 2.5.2 IL‐DLLME procedure

The 5‐mL sample extract was transferred into a 15‐mL conical bottom centrifuge tube, and 0.4 mL of methanol containing 60 μL of [C6MIM][PF6] was quickly injected into the sample extract and the tube was shaken immediately for 30 s and allowed to stand for another 40 s prior to centrifugation at 2795 *g* for 5 min. The fine droplets of the ionic liquid will settle at the bottom of the centrifuge tube. The upper aqueous phase is discarded, the remaining ionic liquid phase is diluted with 200 μL of DMSO, and 5 μL of the diluted ionic liquid phase is injected into the LC–MS/MS system (Wang et al., 2015).

### 2.6 LC–MS/MS analysis

ExionLC™ Series ultra‐high‐performance liquid chromatographic system coupled with the QTRAP® 6500+ MS system from AB Sciex was used for LC–MS/MS analysis. LC–MS/MS system was controlled by Analyst® Software version 1.7 and the results were processed using MultiQuant™ 3.0.2 Software. ZORBAX Eclipse plus C18 (2.1 × 150 mm, 5 μm) analytical column was used for separation and the column oven was set at 40°C. The elution gradient, mobile phase A (0.1% formic acid in LC–MS grade water) and mobile phase B (0.1% formic acid in LC–MS grade methanol/acetonitrile [50/50]) was set up as follows: (1) 0 min, 90% A; 5 min 90% A; 3.0 min, 50% A; 3.5 min 10% A; 5.0 min, 10% A; 5.1 min 2% A; and 7.5 min, 2% A.

This gradient was re‐equilibrated to 90% A for 2.49 min after each run. The flow rate was 0.5 mL/min, and the injection volume was 5 μL. The set optimum electrospray ionization parameters are shown in Table 2 and the compound‐dependent MRM (multiple reaction monitoring) parameters including declustering potential (DP), collision energy (CE), and cell exit potential (CXP) optimized are shown in Table 3.

**TABLE 2:** Mass spectrometry ion source parameters.

Ion source and gas parameters	Optimal value (positive/negative mode)
Ion source voltage (IS)	+5500/−4500
Temperature (Temp)	500
Ion source gas 1 (GS1)	60/50
Ion source gas 2 (GS2)	70/60
Curtain gas (CUR)	40/40
Collision gas (CAD)	Medium/Medium
Entrance potential (EP)	10/−10
Dwell time (ms)	15

**TABLE 3:** Compound‐dependent MRM parameters.

Analyte	Internal standard	Molecular mass	Precursor ion (*m/z*)	Product ions (*m/z*)	DP (volts)	CE (volts)	CXP (volts)
TBZ	ABZ methyl‐D_3_	201	202.3	175/131	131	35/45	20/16
TBZ‐OH	ABZ methyl‐D_3_	217	218.1	191.1/147.1	90	31/41	4/4
ABZ‐SO_2_ NH_2_	ABZ methyl‐D_3_	239	240.1	133.15/198.1	80	27/37	14/22
ABZ‐SO	ABZ methyl‐D_3_	281	282.1	190.1/158.7	126	34/45	10/10
ABZ‐SO_2_	ABZ methyl‐D_3_	298	298.1	266.2/159.07	151	27/37	14/22
FBZ	FBZ‐D_3_	299	300.0	267.9/131	126	29/47	3218
TCZ	TCZ D_3_	258	359.0	274/343.3	176	45/43	14/8
TCZ‐SO	TCZ D_3_	374	375.0	313/356.9	176	45/43	14/8
TCZ‐SO_2_	TCZ D_3_	390	391.0	242.2/312.1	176	45/43	14/8
MBZ‐NH_2_	MBZ‐D_3_	237	238.1	105.1/77.1	80	46/46	4/4
LEVA	FLU‐D_3_	204	205.4	178/91	100	29/37	20/14
OBZ	ABZ methyl‐D_3_	249	250.1	176/148	146	37/49	20/18
MBZ‐OH	MBZ‐D_3_	297	298.3	266/220	90	23/46	4/4
FLU‐NH_2_	FLU‐D_3_	255	256.1	123.3/133.1	71	46/36	4/4
ABZ	ABZ methyl‐D_3_	265	266.1	234/191	131	27/45	26/22
FLU	FLU‐D_3_	313	314.0	281.9/123	176	31/45	14/14
MBZ	MBZ‐D_3_	295	296.0	264/105	131	29/45	14/12
OXY	TCZ D_3_	401	399.9	175.7/201.6	−126	−32/−32	−2/−4
RAF	–	625	623.8	126.9/217	−91	−44/−44	−2/−2
NITRO	TCZ D_3_	300	289.0	126.8/161.7	−99	−18/−18	−4/−4
MBZ‐D_3_	–	289	299.1	263.9/105	161	29/45	32/12
FBZ‐D_3_	–	302	303.1	158.9/267.9	136	59/29	12/54
ABZ methyl‐D_3_	–	268	269.1	190.9/159	126	45/55	22/16
FBZ‐D_3_	–	316	317.1	281.9/123	146	31/47	14/6
TCZ D_3_	–	362	363.1	197/343.3	176	45/43	14/8
FLU‐D_3_	–	316	317.1	281.9/123.0	144	45/55	32/12

### 2.7 Optimization of the extraction method

In the extraction method using IL‐DLLME, the main factors that affect extraction recovery (ER) are as follows: suitable extraction and dispersive solvents; appropriate volume of extraction and dispersive solvents; pH; and salt (Hwang et al., 2018). The optimized factors that affect extraction recovery are extraction solvent ([C6MIM][PF6]), disperser solvent (methanol), volume of [C6MIM][PF6] extraction solvent (60 μL), volume of methanol disperser solvent (0.4 mL), pH of aqueous phase (pH 7), effect of addition of NaCl (No effect), effect of extraction time (40 s), and effect of the centrifugation time at 2795 *g* for 5 min. Extraction recovery (ER) played a major role in optimization of the extraction method. Extraction recovery (ER) is defined as the percentage of total analyte amount (*n*_0_) extracted to the sediment phase (*n*_sed_), ER = *n*_sed_/*n*_0_ × 100 (Scheme, 2021).

## 3 RESULTS AND DISCUSSION

### 3.1 Optimization of the extraction method

#### 3.1.1 Selection of the ionic liquid

The IL‐DLLME procedure is a ternary‐based solvent system, which utilizes miniaturized solvents. A suitable extraction solvent in IL‐DLLME is of paramount importance for the efficient recovery of the analytes of interest from a variety of matrices (Hwang et al., 2018). Nowadays, the trend is to use green (environmentally friendly) solvents for the extraction of analytes. The green extraction solvents used in this work are ionic liquids (ILs) such as [C4MIM][PF6], [C6MIM][PF6], and [C8MIM][PF6]. The aqueous blank liver extracts fortified at MRL concentrations and injected with 0.300 mL of disperser solvent (methanol) containing 50 μL of ionic liquids ([C4MIM][PF6], [C6MIM][PF6], and [C8MIM][PF6]) for each ionic liquid, 10 aqueous extracts fortified with anthelmintic drugs were used (*n* = 10). The solubility of [C4MIM][PF6], [C6MIM][PF6], and [C8MIM][PF6] in water was 18.8, 7.5, and 2.0 mg L^−1^, respectively (Ionic Liquid, 2022). According to the experimental results, the solubility of ionic liquids in water affected their extraction recoveries. An increase in extraction recoveries was observed with an increase in the alkyl chain length of imidazole‐based ionic liquids and the highest recoveries were achieved with imidazole‐based ionic liquids containing six or more alkyl chain lengths. Poor extraction was observed with the [C4MIM][PF6] ionic liquid in comparison with those with the longer alkyl chain length (six to eight alkyl chain lengths). The higher the solubility of the ionic liquid in water, the lower the extraction effect. Figure 2 shows that [C6MIM][PF6] was found to be the best extraction solvent among the three ionic liquids evaluated and it was, therefore, used in all subsequent experiments.

#### 3.1.2 Selection of disperser solvent

A disperser solvent plays an essential role in the formation of a cloudy solution in the IL‐DLLME extraction method. It is important that a suitable solvent is identified for the extraction process. The disperser solvent must be miscible with the extraction solvent (organic phase) and also with the sample solution (aqueous phase) to ensure the formation of a cloudy solution (Hwang et al., 2018). In this study, acetone, methanol, and acetonitrile were evaluated as possible disperser solvents. The study was carried out by fortifying the blank extracts with anthelmintic drug standard mixture at MRL concentrations with 0.300 mL of each disperser solvent (methanol, acetone, and acetonitrile) containing 50 μL of the selected extraction solvent [C6MIM][PF6] and analyzed in LC–MS/MS to calculate the extraction recoveries. Figure 3 shows the results of the disperser solvent optimization. These three solvents all performed well as disperser solvents with extraction recoveries >45% for all analytes. All the disperser solvents produced a cloudy solution when injected into the aqueous phase but methanol was more cloudy as compared to others; however, the highest extraction recovery (>80%) of all the anthelmintic drugs was observed using methanol compared with the other two solvents and this is probably due to its higher compatibility of methanol with aqueous solution than acetone and acetonitrile. Methanol was, therefore, selected for all subsequent experiments; in addition, it was greener than acetonitrile.

#### 3.1.3 Determination of volume of [C6MIM][PF6]

For the determination of the optimum volume of the ionic liquid [C6MIM][PF6], the disperser solvent was kept constant at (0.3 mL) containing different volumes of [C6MIM][PF6] (30, 40, 50, 60, 70, 80, 90, and 100 μL). Results showed that the extraction recoveries of the anthelmintic drug residues increased when the volume of [C6MIM][PF6] was increased from 30 to 70 μL, and then declined from 80 μL with an increase in the volume of IL (Figure S3). When the volume of the ionic liquid is small, the extraction is not efficient, the ionic liquid was not enough to be dispersed in the aqueous phase. In addition, when the ionic liquid volume was high, more of the ionic liquid will sediment after centrifugation, this will result in the dilution of the analyte content in the sedimented ionic liquid. The extraction recovery with [C6MIM][PF6] at 60 μL was above 87% on average of all anthelmintic drugs. Therefore, a volume of 60 μL of [C6MIM][PF6] was selected for all subsequent experiments.

#### 3.1.4 Determination of the optimum volume of disperser solvent

The disperser volume was determined with the volume of [C6MIM][PF6] maintained constant at 60 μL and varying the volume of the disperser solvent (methanol) at 0.2, 0.3, 0.5, 0.6, 0.8, and 1.0 mL. When a small volume of disperser solvent was used, the cloudy solution formed was less tense, hence lower extraction recoveries. The results showed that the best extraction recoveries (ERs) of all anthelmintic drug residues were found to be at volumes of around 0.3 and 0.4 mL and then declined with an increase in the volume of methanol from 0.5 to 1 mL. When a large volume of disperser solvent was used, the ionic liquid could be dissolved in the disperser solvent, resulting in low volume of sedimented ionic liquid phase or no sedimented ionic liquid phase after centrifugation (Wang Gao et al., 2016). The results showed that the highest ERs of the anthelmintic drug residues were obtained when the disperser solvent volume was 0.4 mL. Thus, a volume of 0.4 mL of methanol was selected for all subsequent experiments (Figure S4).

#### 3.1.5 Determination of the optimum pH of aqueous phase

The anthelmintic drugs mixture solution to be extracted was adjusted to pH 2.0–9.0 with hydrochloric acid or ammonium hydroxide prior to the IL‐DLLME procedure (Figure 4). The results showed that the extraction recoveries >80% were fairly constant between pH 5 and 7 and decreased with an increase in pH. Most of the anthelmintic drug compounds are amphoteric, having the potential to act both as an acid and a base. Because most of the compounds are amphoteric, the variation of the pH of the aqueous sample extract had a slight positive impact on extraction recoveries with a slight deviation from pH 7, but with a higher impact at low pH values (2–4) and high pH values (8 and 9). The pH was found to have a minimal positive impact, and hence in this study, the pH of the aqueous extracts was maintained at pH 7.

#### 3.1.6 Effect of the addition of salt (NaCl)

In this work, the disperser solvent at 0.4 mL containing 60 μL of [C6MIM][PF6] was injected in aqueous liver extracts fortified with anthelmintic drug at MRL concentrations, the extracts contained various amounts of NaCl salt (0%, 0.5%, 1%, 2%, 4%, 6%, 8% [w/v]) dissolved by vortex mixing. The results showed an increase of 4% from 90% to 94% (on average for all analytes) in the extraction recoveries of anthelmintic drugs at NaCl concentrations of between 0% and 0.5%, but extraction recoveries decreased to 84% when NaCl concentrations were at 1.0% and further decreased with increase in NaCl percentage (Figure S5). The salt addition (NaCl) in an extraction system can improve the extraction efficiency due to the salting out effect, but when the percentage of the salt increases, the ionic strength in the system increases, and the high ionic strength in an IL‐DLLME system can enhance the solubility of an ionic liquid in aqueous phase, consequently decrease the extraction performance (Wang et al., 2015). The addition of NaCl was, therefore, not used for the subsequent work due to its minimal impact on the extraction recovery and to minimize the extraction time.

#### 3.1.7 Effect of extraction time

In an IL‐DLLME procedure, the maximum quantity of analyte was transferred into the IL phase when the extraction equilibrium is obtained (Wang et al., 2015). The work was carried out by fortifying the aqueous blank extracts with anthelmintic drug standard mixture at MRL and shaking the mixture for 1 min. The effect of extraction time on the yield was evaluated by varying the extraction times (3, 5, 10, 20, 30, 40, 50, and 60 s) prior to centrifugation (Figure 5). The results showed that an extraction recovery of 77% for the anthelmintic drugs was observed when the extraction time was 20 s. To ensure maximum extraction efficiency, the cloudy sample solution was obtained at 40 s prior to centrifugation. Forty seconds was, therefore, selected for all subsequent experiments in this study.

#### 3.1.8 Effect of the centrifugation time

The cloudy solution formed is centrifuged and the fine IL droplets settle at the bottom of the centrifuge tube; however, it is important to establish the optimum centrifugation time with respect to the settlement IL volume and the analyte concentration in the IL phase (Wang et al., 2015). The work was carried out by fortifying the aqueous blank extracts with anthelmintic drug standard mixture at MRL. The cloudy solutions were allowed to stand for 40 s prior to centrifugation at varying times (1, 3, 5, 10, and 15 min) and at 2795 *g*. Results showed that the extraction recoveries >77% of anthelmintic drugs were achieved within the centrifugation time of 5 min (Figure S6). Therefore, a centrifugation time of 5 min for the formed cloudy solution was selected for all subsequent experiments.

### 3.2 Method validation

The extraction method was validated according to Commission Implementing Regulation (EU) 2021/808 of 22 March 2021 and ISO/IEC 17025:2017 (Wang et al., 2015). The validation parameters carried out were specificity/selectivity, LOD, LOQ, linearity, decision limit (CCα), within laboratory reproducibility, and repeatability. The validation study was carried out using seven independent blank liver samples fortified at 0.25, 1.0, and 1.5 times MRL levels of individual analytes, and the experiments were repeated twice on different days bringing the total number of samples to 20 per fortification level (*n* = 21). The samples were quantified with pre‐extraction spiked matrix standards (PrEMS) fortified at 0.0, 0.25, 1.0, 1.5, and 2.0 times MRLs. The calculated LOD, LOQ, coefficient of determination (*R* ^2^), and CCα values based on interbatch reproducibility data are given in Table 4.

**TABLE 4:** Calculated LOD, LOQ, coefficient of determination (*R* ^2^), and CCα values based on interbatch reproducibility data.

Analyte	Fortification level (μg/kg), MRL	Retention time (min)	Mean recovery (μg/kg)	RSD (CV %)	CCα (μg/kg)	Coefficient of determination (*R* ^2^)	LOD (μg/kg)	LOQ (μg/kg)
ABZ	1000	4.20	999.9	11.4	1018.7	.99993	16.6	55.4
ABZ‐SO_2_ NH_2_	1000	2.30	991.4	7.9	1004.3	.99990	20.7	69.0
ABZ –SO	1000	3.26	964.3	7.5	976.6	.99985	26.1	87.0
ABZ‐SO_2_	1000	3.62	1014.6	4.6	1022.0	.99995	16.6	55.4
FBZ	500	4.39	496.3	3.4	501.8	.99983	11.9	39.6
TCZ	250	4.74	234.9	6.2	245.1	.99988	6.9	22.9
TCZ‐SO	250	4.63	233.0	8.9	247.6	.99946	12.0	39.9
TCZ‐SO_2_	250	4.62	243.3	3.8	249.6	.99938	11.3	37.7
MBZ	400	4.19	388.6	12.7	409.4	.99980	12.6	42.1
MBZ‐NH_2_	400	3.21	388.1	6.3	398.5	.99978	14.3	47.5
MBZ‐OH	400	3.63	376.4	10.7	394.0	.99979	14.0	46.8
LEVA	100	2.25	101.2	5.5	110.2	.99945	4.4	14.5
OBZ	200	3.61	192.9	8.3	206.5	.99967	9.0	29.9
FLU	400	4.25	384.1	3.15	389.3	.99982	12.7	42.2
FLU‐NH_2_	400	3.34	401.8	10.3	418.0	.99984	11.3	37.7
OXY	500	4.67	456.0	12.8	476.9	.99993	10.0	33.4
RAF	150	6.30	139.7	1.4	141.9	.99965	6.5	21.6
NITRO	20	4.26	18.6	2.8	23.1	.99993	0.37	1.2
TBZ	100	2.48	101.1	7.9	114.0	.99980	3.5	11.6
TBZ‐OH	100	2.26	100.0	4.4	107.1	.99955	4.1	13.6

#### 3.2.1 Linearity

The linearity of the mass spectrometry using a 5‐point calibration curve in the range of 0.0, 0.5, 1.0, 1.5, and 2.0 times MRLs of compounds was achieved (Figure S1). The coefficient of determination (*r* ^2^) values for the calibration curves used in the study were ≥.99 and the *r* ^2^ values ranged from .9980 to .9999 as listed in Table 4.

#### 3.2.2 Selectivity/specificity

**FIGURE 1:**
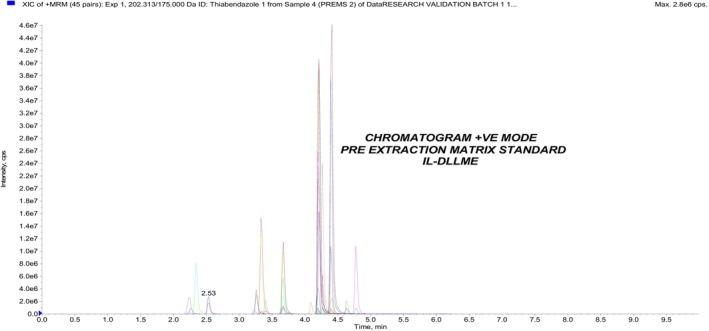
Chromatogram of a blank liver sample fortified at MRLs.

In this experiment, 10 different lots of caprine and 10 different lots of ovine blank samples were analyzed together with 10 different lots of caprine and 10 different lots of ovine blank samples fortified at MRL concentrations with the anthelmintic drugs standard to check for any interferences of signals, peaks or ion traces in the region of interest where the target analyte is expected to elute. The fortified liver and blank liver samples were analyzed using mass spectrometry (Figure S2). The anthelmintic drug standards fortified at MRLs were extracted using IL‐DLLME and analyzed using LC–MS/MS. A typical ion chromatogram is shown in Figure 1. No interfering peaks were observed at the retention time for the respective anthelmintic analytes.

**FIGURE 2:**
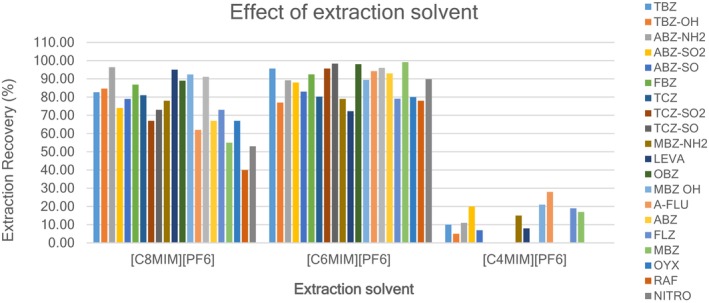
Effect of ionic liquid extraction solvents on the recoveries of 20 anthelmintic drugs (blank extracts, 5.0 mL; ionic liquid ([C4MIM][PF6], [C6MIM][PF6], [C8MIM][PF6]), 5 μL; disperser solvent (acetonitrile), 0.3 mL).

**FIGURE 3:**
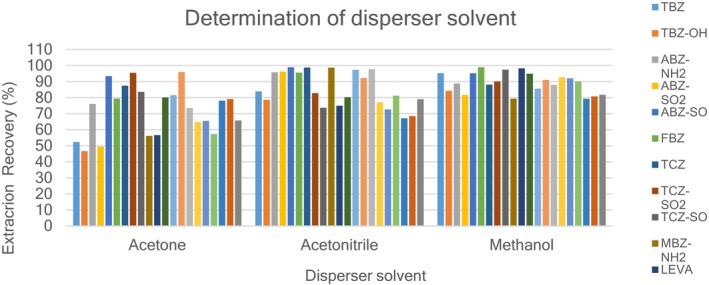
Effect of disperser solvent on extraction recoveries of 20 anthelmintic drugs (blank extracts, 5.0 mL; ionic liquid [C6MIM][PF6] 50 μL; disperser solvent (methanol, acetonitrile, acetone), 0.3 mL).

**FIGURE 4:**
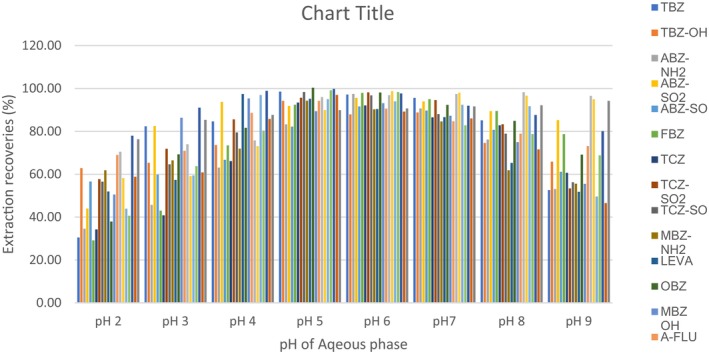
Effect of pH of aqueous blank extracts on extraction recoveries of 20 anthelmintic drugs (blank extracts, 5.0 mL (pH 2, 3, 4, 5, 6, 7, 8, and 9); ionic liquid [C6MIM][PF6], 60 μL; disperser solvent (methanol), 0.4 mL).

**FIGURE 5:**
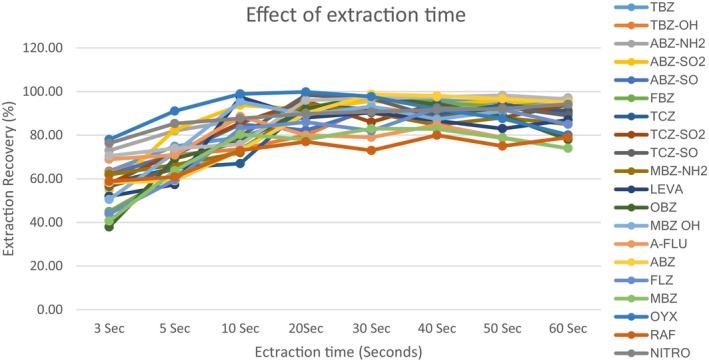
Effect of extraction time on extraction recoveries of 20 anthelmintic drugs (spiked aqueous blank extracts, 5.0 mL with 400 μL containing 60 μL of [C6MIM][PF6]) left to stand for 3, 5, 10, 20, 30, 40, 50, and 60 s after shaking.

#### 3.2.3 Trueness/accuracy

Trueness means the closeness of agreement between the average value obtained from a large series of test results and an accepted reference value. For the determination of accuracy/trueness, caprine and ovine liver samples were fortified at 0.25 × MRL, 1.0 × MRL, and 1.5 × MRL for each anthelmintic compound. Mean recovery (*n* = 21) for all analytes, determined on three separate occasions, was within the recommended range of 70%–120% for values >1 to 10 μg/kg and 80%–120% for spiking values <10 μg/kg as in Commission Implementing Regulation (EU) 2021/808 of 22 March 2021. The percentage recoveries for all the compounds are shown in Table 6 below.

#### 3.2.4 The decision limit (CCα)

The decision limit for confirmation (CCα) means the limit at and above which it can be concluded with an error probability of *α* that a sample is noncompliant and the value 1 − *α* means statistical certainty in percentage that the permitted limit has been exceeded (Commission, 2021). The CCα values were determined by analyzing at least 21 blank materials per matrix fortified with the analyte (s) at MRL concentrations. The mean concentration at MRL plus 1.64 times the corresponding standard deviation equals the decision limit (*α* = 5%). Table 4 above shows the CCα values for the anthelmintic drugs, using the interbatch reproducibility data.

#### 3.2.5 Precision (repeatability and reproducibility)

Precision refers to the closeness of agreement between independent test results obtained under stipulated (predetermined) conditions and precision is the repeatability and reproducibility of the method. Repeatability refers to the closeness of measured values between repeated measurements of identical test items carried out under identical conditions, same method on identical test items in the same laboratory by the same operator using the same equipment. Reproducibility refers to the closeness of measured values between repeated measurements obtained under changed conditions (time, operators, laboratory, reagents; Commission, 2021). In this study, validation was carried out using different operators but in the same laboratory. For the determination of repeatability/reproducibility, bovine liver samples were fortified at 0.25, 1.0, and 1.5 times MRL for each anthelmintic compound. The experiment was repeated twice by two different analysts for reproducibility purposes. The coefficient of variation (CV) values for the repeated analysis of PrEMS, under reproducibility conditions calculated, are within the reproducibility range given in Table 5 below adapted from the Horwitz Equation CV = 2^(1−0.5 log C)^ (where CV is the between‐laboratory coefficient of variation expressed as percent and *C* is the concentration of analyte found or added, expressed as a mass fraction [negative powers of 10]) and ranged from 1.7% to 16.9%. The coefficient of variation (CV) values for the repeated analysis of PrEMS, under repeatability conditions calculated, are within the repeatability range given in Table 5 below which are two thirds of the CVs adapted from the Horwitz Equation CV = 2^(1–0.5 log C)^ and ranged from 1.7% to 16.1%. The calculated repeatability CVs and reproducibility CVs are listed in Table 6 below.

**TABLE 5:** Examples of reproducibility CVs for quantitative methods over a range of analyte mass fractions [10].

Mass fraction	Mass fraction reproducibility CV (%)
>1000 μg/kg	16 (adapted from Horwitz equation)
>120–1000 μg/kg	22 (adapted from Horwitz equation)
10–120 μg/kg	25*
<10 μg/kg	30*

* The CV (%) presented is a guideline and should be as low as reasonably possible.

**TABLE 6:** Intra‐ and interbatch variation for recovery of anthelmintic drug residues from liver samples.

Analyte	Fortification level (μg/kg)	Mean recovery (%)	Within batch (repeatability), % CV	Acceptable adopted repeatability range	Reproducibility % CV	Acceptable adopted reproducibility range
ABZ	250	91.8	5.9	≤14.7%	5.7	≤22%
	1000	98.7	11.4		11.4	
	1500	99.5	5.9	≤10.7%	5.9	≤16%
ABZ‐SO_2_ NH_2_	250	94.2	1.7	≤14.7%	7.1	≤22%
	1000	98.5	7.9		8.0	
	1500	100.5	7.7	≤10.7%	7.9	≤16%
ABZ –SO	250	96.2	5.2	≤14.7%	10.3	≤22%
	1000	96.4	7.5		7.6	
	1500	98.9	8.8	≤10.7%	8.5	≤16%
ABZ‐SO_2_	250	87.3	13.9	≤14.7%	13.8	≤22%
	1000	99.9	6.3		4.6	
	1500	95.3	8.5	≤10.7%	8.7	≤16%
FBZ	125	96.9	4.8	≤14.7%	3.9	≤22%
	500	96.4	7.5		3.4	
	750	97.8	12.0		11.8	
TCZ	62.5	95.3	9.6	≤16.7%	9.7	≤25%
	250	94.0	13.4	≤14.7%	6.2	≤22%
	375	86.8	14.2		5.6	
TCZ‐SO	62.5	88.8	11.6	≤16.7%	4.6	≤25%
	250	93.2	8.7	≤14.7%	9.0	≤22%
	375	90.6	12.3		12.4	
TCZ‐SO_2_	62.5	89.9	11.7	≤16.7%	11.4	≤25%
	250	97.3	10.2	≤14.7%	3.8	≤22%
	375	91.2	13.0		7.9	
MBZ	100	99.4	10.1	≤14.7%	4.7	≤22%
	400	97.1	12.7		12.7	
	600	96.2	14.3		5.5	
MBZ‐NH_2_	100	93.9	13.8	≤14.7%	16.9	≤22%
	400	97.0	9.3		6.3	
	600	94.6	11.7		11.8	
MBZ‐OH	100	100.2	8.4	≤14.7%	2.7	≤22%
	400	94.1	10.3		10.7	
	600	98.3	9.6		6.8	
LEVA	25	91.8	4.0	≤16.7%	0.47	≤25%
	100	101.6	5.5		5.5	
	150	101.2	5.3	≤14.7%	1.6	≤22%
OBZ	50	105.0	8.1	≤16.7%	8.51	≤25%
	200	96.5	7.9	≤14.7%	8.27	≤22%
	300	86.7	7.77		9.42	
FLU	100	97.6	9.9	≤16.7%	3.8	≤25%
	400	88.5	12.5	≤14.7%	3.2	≤22%
	600	83.1	6.2		6.1	
FLU‐NH_2_	100	85.6	16.1	≤16.7%	16.5	≤25%
	400	99.8	9.9	≤14.7%	10.3	≤22%
	600	95.9	8.8		3.1	
OXY	125	87.7	7.66	≤14.7%	4.78	≤22%
	500	91.2	12.2		12.8	
	750	87.8	9.9		0.43	
RAF	37.5	98.7	9.9	≤16.7%	10.5	≤25%
	150	93.1	10.8	≤14.7%	1.4	≤22%
	225	86.2	13.2		13.2	
NITRO	5	83.1	7.8	≤20%	8.1	≤30%
	20	92.9	8.8	≤16.7%	2.9	≤25%
	30	91.2	11.8		12.1	
TBZ	25	87.4	2.3	≤16.7%	7.1	≤25%
	100	100.8	7.5		7.9	
	150	83.2	7.3	≤14.7%	7.7	≤22%
TBZ‐OH	25	88.9	10.1	≤16.7%	3.3	≤25%
	100	100.0	4.9		4.4	
	150	98.8	6.1	≤14.7%	8.2	≤22%

#### 3.2.6 Comparison of IL‐DLLME method with QuEChERS method

The IL‐DLLME method was compared to QuEChERS standard method for confirmation of benzimidazole drug residues in bovine liver (Table 7). The QuEChERS method was validated and accredited by the Southern African Development Community Accreditation Services (SADCAS). This method also passed the interlaboratory proficiency testing between Botswana National Veterinary Laboratory and FERA Science Ltd, Fapas® proficiency testing provided by FERA Science Ltd, U.K, as well as Progetto Trieste proficiency testing (Italy). The two methods were compared by the recovered concentration results spiked at 1.0 times MRL and extracted with IL‐DLLME and QuEChERS method. The *t*‐values of the results were calculated using a two‐tailed paired test at the 95% confidence level. Nitroxinil (NITRO), oxyclozanide (OXY), and rafoxanide (RAFO) were not validated on the QuEChERS method and the results obtained for these three analytes using IL‐DLLME could, thus, not be compared. The *t*‐values for all the analytes that are comparable are below the *t*‐critical value (Table 7); this finding shows that the method is reproducible and gives reliable results. The two methods were also compared in terms of resources, time, and costs (Table 8). IL‐ DLLME method proved to be cheaper in terms of costs of reagents used and the quantity of waste reagents.

**TABLE 7:** Comparison of IL‐DLLME method with QuEChERS method.

Analyte	Method	Recovered concentration, 1.0 MRL	*t*‐Value	*t*‐Critical value
ABZ	IL‐DLLME	889	−1.36	−4.30
	QuEChERS	912		
ABZ‐SO_2_ NH_2_	IL‐DLLME	960	1.38	4.30
	QuEChERS	910		
ABZ –SO	IL‐DLLME	934	0.53	4.30
	QuEChERS	874		
ABZ‐SO_2_	IL‐DLLME	899	−0.81	−4.30
	QuEChERS	979		
FBZ	IL‐DLLME	436	−0.98	−4.30
	QuEChERS	484		
TCZ	IL‐DLLME	239	−0.71	−4.30
	QuEChERS	233		
TCZ‐SO	IL‐DLLME	240	1.14	4.30
	QuEChERS	220		
TCZ‐SO_2_	IL‐DLLME	238	1.07	4.30
	QuEChERS	228		
MBZ	IL‐DLLME	397	−0.59	−4.30
	QuEChERS	397		
MBZ‐NH_2_	IL‐DLLME	377	1.05	4.30
	QuEChERS	357		
MBZ‐OH	IL‐DLLME	381	−0.61	−4.30
	QuEChERS	361		
LEVA	IL‐DLLME	89	−0.22	−4.30
	QuEChERS	95		
OBZ	IL‐DLLME	185	0.77	4.30
	QuEChERS	184		
FLU	IL‐DLLME	384	−0.38	−4.30
	QuEChERS	389		
FLU‐NH_2_	IL‐DLLME	368	0.46	4.30
	QuEChERS	371		
OXY	IL‐DLLME	438	–	–
	QuEChERS	–		
RAF	IL‐DLLME	8.7	–	–
	QuEChERS	–		
NITRO	IL‐DLLME	16.6	–	–
	QuEChERS	–		
TBZ	IL‐DLLME	94	0.56	4.30
	QuEChERS	91		
TBZ‐OH	IL‐DLLME	91	0.45	4.30
	QuEChERS	88		

**TABLE 8:** Comparison of IL‐DLLME and QuEChERS method in terms of time, costs, and resources.

Time, costs, and resources	QuEChERS	IL‐DLLME
Weighing (20 samples)	1 h	1 h
Time of weighing of salts	2 h	–
Extraction time (20 samples)	1.5 days	1 day
Centrifugal time	10 min + 5 min = (15 min)	10 min + 5 min = (15 min)
Volume of acetonitrile (extraction)	12 mL	8 mL
Extraction solvent	dSPE (7 mL)	0.06 mL
Volume of methanol	0.4 mL	0.4 mL
Reagents going to waste (20 samples)	Acetonitrile (100 mL) MgSO_4_ (120 g) C18 sorbent (10 g)	Acetonitrile 60 mL
Drying time (20 samples)	2 h (has H_2_0)	1 h
NaCl per sample	1 g	–
MgSO4 per sample	5 g solvent extraction + 1 g dispersive SPE	–
C18 sorbent per sample	500 mg	–
[C6MIM][PF6] per sample	–	0.06 mL
DMSO	0.2 mL	0.2 mL
Estimated costs of reagents/year at Botswana National Veterinary Laboratory	2 × 200G C18 sorbent (800 USD) 3 × 500 g MgSO4 (500 USD) 1 × 250 g NaCl (70 USD) Estimated total costs (1370 USD)	1 × 50 g [C6MIM][PF6] (1044 USD) Enough to be used for 2 years

#### 3.2.7 Application of IL‐DLLME LC–MS/MS method on real samples

The IL‐DLLME/LC–MS/MS method was applied to 32 small stock (18 caprine and 14 ovine) liver samples received from municipal abattoirs at Botswana National Veterinary Laboratory for anthelmintic drug residue analysis. The results obtained showed that the anthelmintic drug residues were all below the detection capability, and therefore, the samples were passed as fit for human consumption.

## 4 CONCLUSIONS

The reported IL‐DLLME/LC–MS/MS method was developed, optimized, and validated for detecting and monitoring anthelmintic drug residues in meat. The method employs a simple, fast, sensitive, cost‐effective, and environmentally friendly IL‐DLLME extraction technique. The developed method was validated in accordance with Commission Implementing Regulation (EU) 2021/808 of 22 March 2021 and all validated parameters comply with the set standards. The developed and validated method was comparable to QuEChERS standard method with respect to reliable, precise, sensitive, and reproducible and was found not to be significantly different. In addition, the method was found to be cheap to use and fit for the intended purpose.

## AUTHOR CONTRIBUTIONS

**Rebagamang Tshepho:** Formal analysis (lead); investigation (lead); methodology (lead); validation (lead); writing – original draft (equal); writing – review and editing (equal). **Simiso Dube:** Conceptualization (equal); project administration (supporting); supervision (equal); writing – review and editing (equal). **Mathew M. Nindi:** Conceptualization (equal); funding acquisition (equal); project administration (equal); resources (lead); supervision (equal); writing – review and editing (equal).

## FUNDING INFORMATION

The research was partially supported by UNISA Postgraduate bursary and IAEA coordinated research project that provided labeled standards and ionic liquids (Grant # CRP D52041 BOS22139).

## CONFLICT OF INTEREST STATEMENT

The authors (R Tshepho, S. Dube, and M.M Nindi) declare that they have no potential conflicts of interest in the authorship or publication of this contribution, the research does not involve human participants and/or animals, all the authors that contributed to this work have consented.

## ETHICS STATEMENT

No humans were involved in this study. All the authors have Ethical Clearance Certificates for nonhuman/animal involvement research.

## PERMISSION TO REPRODUCE MATERIAL FROM OTHER SOURCES

No material was reproduced from other sources.

## Supporting information


Figure S1.

Figure S2.

Figure S3.

Figure S4.

Figure S5.

Figure S6.

## Data Availability

The data that support the findings of this study are available from the corresponding author upon reasonable request.
