# Supplementary material for: Ionic liquid‐based dispersive liquid–liquid microextraction of anthelmintic drug residues in small‐stock meat followed by LC‐ESI‐MS/MS detection
Source: Food Sci Nutr. 2023 Jul 22;11(10):6288–302. doi: 10.1002/fsn3.3568 (PMC10563727; doi:10.1002/fsn3.3568)
Supplement: Supplementary file 1 — Figure S1. [file FSN3-11-6288-s001.docx]

Supplementary 1

**Figure S1:** Chromatogram of a blank liver sample
