# Supplementary material for: Ionic liquid‐based dispersive liquid–liquid microextraction of anthelmintic drug residues in small‐stock meat followed by LC‐ESI‐MS/MS detection
Source: Food Sci Nutr. 2023 Jul 22;11(10):6288–302. doi: 10.1002/fsn3.3568 (PMC10563727; doi:10.1002/fsn3.3568)
Supplement: Supplementary file 3 — Figure S3. [file FSN3-11-6288-s005.docx]

**Figure S3:** Effect of volume of IL on extraction recoveries of 21 anthelmintic drugs (blank extracts, 5.0 mL; ionic liquid [C6MIM][PF6] (30, 40, 50, 60, 70, 80, 90, 100 μL); disperser solvent (methanol), 0.3 mL)
