# Supplementary material for: Ionic liquid‐based dispersive liquid–liquid microextraction of anthelmintic drug residues in small‐stock meat followed by LC‐ESI‐MS/MS detection
Source: Food Sci Nutr. 2023 Jul 22;11(10):6288–302. doi: 10.1002/fsn3.3568 (PMC10563727; doi:10.1002/fsn3.3568)
Supplement: Supplementary file 4 — Figure S4. [file FSN3-11-6288-s002.docx]

**Figure S4:** Effect of disperser solvent on extraction recoveries of 21 anthelmintic drugs (blank extracts, 5.0 mL; ionic liquid [C6MIM][PF6] 60 μL; disperser solvent (methanol), (0.2, 0.3, 0.4, 0.5, 0.6, 0.8, 1.0 mL)
