# Supplementary material for: Ionic liquid‐based dispersive liquid–liquid microextraction of anthelmintic drug residues in small‐stock meat followed by LC‐ESI‐MS/MS detection
Source: Food Sci Nutr. 2023 Jul 22;11(10):6288–302. doi: 10.1002/fsn3.3568 (PMC10563727; doi:10.1002/fsn3.3568)
Supplement: Supplementary file 5 — Figure S5. [file FSN3-11-6288-s006.docx]

**Figure S5:** Effect of addition of NaCl on extraction recoveries of 21 anthelmintic drugs (blank extracts, 5.0 mL with NaCl (0, 0.5, 1.0, 2.0, 4.0, 6.0, and 8.0%); ionic liquid [C6MIM][PF6], 60 μL; disperser solvent (methanol), 0.4 mL)
