# Supplementary material for: Ionic liquid‐based dispersive liquid–liquid microextraction of anthelmintic drug residues in small‐stock meat followed by LC‐ESI‐MS/MS detection
Source: Food Sci Nutr. 2023 Jul 22;11(10):6288–302. doi: 10.1002/fsn3.3568 (PMC10563727; doi:10.1002/fsn3.3568)
Supplement: Supplementary file 6 — Figure S6. [file FSN3-11-6288-s004.docx]

**Figure S6:** Effect of centrifugation time on extraction recoveries of 21 anthelmintic drugs (5 mL of spiked aqueous blank extracts with 60 μL of [C6MIM][PF6]) centrifuged at different times (1, 3, 5, 10 and 15 min) after shaking
